# Body composition and arsenic metabolism: a cross-sectional analysis in the Strong Heart Study

**DOI:** 10.1186/1476-069X-12-107

**Published:** 2013-12-09

**Authors:** Matthew O Gribble, Ciprian M Crainiceanu, Barbara V Howard, Jason G Umans, Kevin A Francesconi, Walter Goessler, Ying Zhang, Ellen K Silbergeld, Eliseo Guallar, Ana Navas-Acien

**Affiliations:** 1 Department of Epidemiology and Welch Center for Prevention, Epidemiology and Clinical Research, Johns Hopkins University Bloomberg School of Public Health, 615 N. Wolfe Street Office W7513D, Baltimore MD 21205MD, USA; 2 Department of Biostatistics, Johns Hopkins University Bloomberg School of Public Health, Baltimore, MD, USA; 3 MedStar Health Research Institute, Hyattsville, MD, USA; 4 Georgetown-Howard Universities Center for Clinical and Translational Science, Washington DC, USA; 5 Institute of Chemistry – Analytical Chemistry, Karl-Franzens University Graz, Graz, Austria; 6 University of Oklahoma Health Sciences Center, Oklahoma City, OK, USA; 7 Department of Environmental Health Sciences, Johns Hopkins University Bloomberg School of Public Health, Baltimore, MD, USA; 8 Department of Medicine, Johns Hopkins Medical Institutions, Baltimore, MD, USA

**Keywords:** American Indians, Arsenic, Arsenic metabolism, Arsenic species, Obesity, Body mass index, Strong Heart Study

## Abstract

**Objective:**

The objective of this study was to evaluate the association between measures of body composition and patterns of urine arsenic metabolites in the 1989–1991 baseline visit of the Strong Heart Study, a cardiovascular disease cohort of adults recruited from rural communities in Arizona, Oklahoma, North Dakota and South Dakota.

**Methods:**

We evaluated 3,663 Strong Heart Study participants with urine arsenic species above the limit of detection and no missing data on body mass index, % body fat and fat free mass measured by bioelectrical impedance, waist circumference and other variables. We summarized urine arsenic species patterns as the relative contribution of inorganic (iAs), methylarsonate (MMA) and dimethylarsinate (DMA) species to their sum. We modeled the associations of % arsenic species biomarkers with body mass index, % body fat, fat free mass, and waist circumference categories in unadjusted regression models and in models including all measures of body composition. We also considered adjustment for arsenic exposure and demographics.

**Results:**

Increasing body mass index was associated with higher mean % DMA and lower mean % MMA before and after adjustment for sociodemographic variables, arsenic exposure, and for other measures of body composition. In unadjusted linear regression models, % DMA was 2.4 (2.1, 2.6) % higher per increase in body mass index category (< 25, ≥25 & <30, ≥30 & <35, ≥35 kg/m^2^), and % MMA was 1.6 (1.4, 1.7) % lower. Similar patterns were observed for % body fat, fat free mass, and waist circumference measures in unadjusted models and in models adjusted for potential confounders, but the associations were largely attenuated or disappeared when adjusted for body mass index.

**Conclusion:**

Measures of body size, especially body mass index, are associated with arsenic metabolism biomarkers. The association may be related to adiposity, fat free mass or body size. Future epidemiologic studies of arsenic should consider body mass index as a potential modifier for arsenic-related health effects.

## Background

Inorganic arsenic (iAs) is a known human carcinogen [1]. Major sources of exposure to inorganic arsenic for the general population include contaminated water and food [2-5]. After exposure, iAs is methylated [6] and then eliminated in urine as iAs (~10-30%), monomethylarsonate (MMA, ~10-20%) and dimethylarsinate (DMA, ~60-80%) [7-12]. Variation in the pattern of urine metabolites has been related to arsenic-associated outcomes including skin lesions [13-15], cancers [16-22] and cardiovascular diseases [23,24]. This may be due to urine arsenic species patterns reflecting differential bioavailability across individuals [25-27] of arsenic species with distinct toxicities [28-31]. Genetic variants partly determine the pattern of urine arsenic metabolites [32]. The main enzyme identified in arsenic methylation reactions is arsenic (III) methyltransferase [33-35] but other enzymes, cofactors, and transporters may also contribute [32].

Non-genetic determinants of arsenic metabolism may include sex, smoking, alcohol intake and nutritional status including dietary folate and vitamin B [36,37]. Recently, increased body mass index has been related to higher %DMA and lower %MMA in urine in populations from Northern Mexico, Central Europe, and Bangladesh [10,38,39], although other studies in Bangladesh have been inconsistent [13,40]. In a study of 303 adolescents from Taiwan, adolescents with obesity had higher %MMA and lower %DMA compared to adolescents without obesity [41], but the differences were not statistically significant. Body mass index is an imperfect measure of adiposity, as it reflects both adipose tissue and fat free mass [42]. Other measures of adiposity or body composition were not evaluated in these studies. Studying the relationships of arsenic metabolism with other measures of adiposity and body composition, such as waist circumference (a measure of central adiposity), body fat and fat free mass, might help to distinguish whether associations are with adiposity or with fat free body mass.

The Strong Heart Study (SHS) is a large rural cohort study with standardized measures of cardiovascular risk factors including multiple measures of body size and body composition (body mass index, percent body fat and fat free mass measured by bioelectrical impedance, and waist circumference). Participants also provided a spot urine sample, allowing for the measurement of arsenic metabolites. The objective of this study was to evaluate the cross-sectional association of adiposity with the pattern of urine arsenic metabolites in SHS participants in 1989–1991.

## Methods

### Study population

The recruitment and examination protocols of the SHS have been described in detail [43]. Briefly, the tribal rolls from 13 American Indian tribes and communities were used to recruit the study population, aiming for 1,500 participants at each study center (Arizona, Oklahoma, and North/South Dakota). A total of 4,549 adults were recruited in 1989–1991. All participants provided informed consent, and all study protocols were approved by institutional and Indian Health Service institutional review boards as well as by the participating tribes.

Total urine arsenic and arsenic species, including iAs, MMA and DMA were measured in 3,974 participants with sufficient urine sample available. For this study, we further excluded one participant missing iAs measurements and participants with iAs (n = 210), MMA (n = 29) or DMA (n = 1) below the limit of detection. We also excluded participants missing data on body mass index (n = 16), percent body fat (n = 53), waist circumference (n = 5), education (n = 4), drinking status (current/ever/never; n = 9) or total urine arsenic (n = 1), leaving 3,663 participants for the analysis. The participants included in the analyses were similar to the overall study population with respect to sociodemographic characteristics (Additional file 1: Appendix 1).

### Measures of body composition

Participants were interviewed and physically examined by centrally trained staff according to a standardized protocol [43-45]. Participants were examined in the morning after a 12-hour overnight fast, which included instruction not to eat breakfast the morning of the visit to the exam, and to eat or drink nothing but water after 9:00 the previous evening [43,45]. Each study center had a designated anthropometry supervisor. Height was measured standing in centimeters rounded to the nearest integer, and weight was measured in kilograms using a scale that was re-zeroed each day and calibrated against a known 50 lb weight every month or whenever the scale was moved. Body mass index was calculated by dividing weight in kilograms by height in meters squared. Waist circumference at umbilicus was measured supine in centimeters rounded to the nearest integer. The bioelectric impedance was measured supine on the right side, unless amputated, using Impedance Meter Model # B1A101 (RJL Equipment Company) [45]. For the impedance measurements, participants were checked by examiners to confirm they had not exercised vigorously for the past 12 hours, had not consumed alcohol in the past 24 hours, and were not dehydrated [45]. We estimated fat free mass and percentage of body fat fat freeby equations based on total body water validated in American Indian populations [46,47].

$$\begin{array}{l} {\mathrm{Water}}_{\mathrm{males}}\left(L\right)=\\ \;e^{\left\{1.1782\times \log\;\left[\mathrm{height}\;\left(\mathrm{cm}\right)\right]-0.5968\times \log\;\left[\mathrm{resistance}\;\left(\Omega \right)\right]+0.3226\times \log\;\left[\mathrm{weight}\;\left(\mathrm{kg}\right)\right]\right\}}.\\ {\mathrm{Water}}_{\mathrm{females}}\left(L\right)=\\ \;e^{\left\{1.2004\times \log\;\left[\mathrm{height}\;\left(\mathrm{cm}\right)\right]-0.5529\times \log\;\left[\mathrm{resistance}\;\left(\Omega \right)\right]+0.2164\times \log\;\left[\mathrm{weight}\;\left(\mathrm{kg}\right)\right]\right\}}.\\ \mathrm{Fat}\;\mathrm{free}\;\mathrm{mass}\;\left(\mathrm{LBM}\right)\;\mathrm{in}\;\mathrm{kilograms}=\mathrm{water}\;\left(L\right)/0.732.\\ \%\;\mathrm{body}\;\mathrm{fat}=\left[\left(\mathrm{weight}-\mathrm{LBM}\right)/\mathrm{weight}\right]*100\% \end{array}$$

### Urine arsenic metabolites

Spot urine samples collected at the 1989–91 baseline visit were stored at -80°C at the Penn Medical Laboratory, MedStar Health Research Institute (Hyattsville, MD and Washington, DC, USA) (Lee et al. 1990). In 2009, aliquots of up to 1.0 mL from each participant were shipped to the Trace Element Laboratory of the Institute of Chemistry-Analytical Chemistry, Karl Franzens University (Graz, Austria) for trace element analysis [48]. The laboratory uses calibration blanks and calibration standards, calibration checks, sample spikes and blanks, NIES No 18 *Human* urine, and in-house urine reference samples for quality control/quality assurance purposes. Concentrations of iAs, MMA, and DMA were determined by anion-exchange high performance liquid chromatography (HPLC; Agilent 1100, Agilent Technologies, Waldbronn, Germany) coupled to inductively coupled plasma mass spectrometry (ICPMS) (Agilent 7700x). The limits of detection were 0.1 μg/L for iAs, MMA, and DMA. The inter-assay coefficients of variation for iAs, MMA, and DMA for the in-house reference urine specimens were 6.0%, 6.5%, and 5.9%.

### Statistical analysis methods

To evaluate arsenic metabolism, we calculated %iAs, %MMA, and %DMA as the relative contribution of iAs, MMA, or DMA to their sum, multiplied by 100, and analyzed the association of each % species biomarker with each measure of body composition (body mass index, percent body fat, fat free mass and waist circumference) separately using linear regression models. Body mass index was categorized as < 25, ≥25 and < 30, ≥30 and < 35 and ≥35 kg/m^2^. Percent body fat, fat free mass and waist circumference were categorized as sex-specific quartiles. Adjusted models accounted for study region, age (< 55 and ≥55 years), sex, smoking status (current/ever/never), drinking status (current/ever/never), high school completion (yes/no), and quartiles of total urine arsenic adjusted for specific gravity. To obtain quartiles of total urine arsenic adjusted for specific gravity (a proxy for urine dilution of the spot urine sample), we fit linear regressions of measured urine arsenic on specific gravity and took quartiles of the residuals. We considered sensitivity analyses adjusting for restricted cubic splines of continuous variables (e.g. age, specific gravity-corrected arsenic) with consistent findings (data not shown). In addition to evaluating each measure of body composition separately, we also contrasted the four measures of body composition in models with multiple measures of body composition. Because both arsenic metabolism and adiposity differ by sex, we evaluated the association between % arsenic species and measures of body composition stratified by sex, with consistent findings (data not shown). Because approximately half of the study population had diabetes [49], we also evaluated the association between % arsenic species and adiposity by diabetes status with consistent findings (data not shown).

The %iAs, %MMA and %DMA are skewed distributions but not log-normal (Additional file 2: Appendix 2), resulting in a poor fit for linear regression models. In a sensitivity analysis, we used generalized gamma regression [50] to evaluate the association between urine arsenic metabolism biomarkers with measures of body composition. The generalized gamma distribution is characterized by a location parameter β that defines the position of the distribution median, a scale parameter σ that reflects the distribution’s spread, and a shape parameter κ that defines the family of the distribution (lognormal, gamma, Weibull, etc.). We also conducted a second sensitivity analysis using beta regression for each % arsenic species (divided by 100) since these biomarkers are proportion data [51] and Dirichlet regression, a multivariate modification of the beta regression that models all % arsenic species as a set that must sum to one [52]. All statistical analyses were performed in Stata/SE 11.2, augmented with < dirifit > and < betafit > contributed packages.

## Results

Mean (SD) body mass index, percent body fat, fat free mass and waist circumference were 30.9 (6.3) kg/m^2^, 36.2 (9.1)%, 53 (11)%, and 105.4 (14.7) cm, respectively. The Spearman correlation coefficient was 0.66 between body mass index and percent body fat, 0.36 between body mass index and fat free mass, 0.88 between body mass index and waist circumference, -0.35 between percent body fat and fat free mass, and 0.63 between percent body fat and waist circumference (Table 1).

**Table 1:** Spearman correlation coefficients of body composition measures and arsenic metabolism biomarkers

	**Body mass index**	**% Body fat**	**Fat free body mass**	**Waist circumference**	**% iAs**	**% MMA**	**% DMA**
**Body mass index**	1.00						
**% Body fat**	0.66	1.00					
**Fat free body mass**	0.36	-0.35	1.00				
**Waist circumference**	0.88	0.63	0.33	1.00			
**% iAs**	- 0.16	-0.25	0.15	-0.16	1.00		
**% MMA**	- 0.32	-0.35	0.07	-0.29	0.47	1.00	
**% DMA**	0.29	0.35	-0.12	0.27	-0.82	-0.87	1.00

N = 3,663 participants.

Median (IQR) were 7.9 (5.6, 11.0)% for %iAs, 13.9 (10.8, 17.5)% for MMA and 77.8 (72.0, 82.7)% for %DMA (Table 2). The %iAs and %MMA biomarkers were moderately positively correlated (Spearman correlation coefficient 0.47), while %DMA was strongly negatively correlated with %iAs and %MMA (Spearman correlation coefficients -0.82 and -0.87, respectively) (Table 1). Median %iAs was higher in men, younger participants, current smokers, and current drinkers, and lower in Oklahoma (Table 2). Median %MMA was higher in men, older participants, participants from North and South Dakota, and current smokers. Median %DMA was lower in men, participants from North and South Dakota, current smokers and current drinkers.

**Table 2:** Arsenic metabolite proportions by participant characteristics

**Participants**	**N (%)**	**% iAs median (IQR)**	**P value**	**% MMA median (IQR)**	**P value**	**% DMA median (IQR)**	**P value**
Overall	3,663	7.9 (5.6, 11.0)		13.9 (10.8, 17.5)		77.8 (72.0, 82.7)	
Sex							
Female	2,157 (58. 9)	7.0 (5.0, 10.0)		13.0 (10.0, 16.0)		79.7 (74.7, 84.1)	
Male	1,506 (41.1)	9.4 (6.7, 12.9)	<0.001	15.7 (12.4, 19.4)	<0.001	74.5 (67.9, 80.0)	<0.001
Age							
< 55 years	1,836 (50.1)	8.5 (6.0, 11.5)		13.9 (10.8, 17.6)		77.3 (71.7, 82.2)	
≥ 55 and < 65 years	1,190 (32.5)	7.6 (5.4, 10.5)		13.6 (10.7, 17.3)		78.6 (72.4, 83.3)	
≥ 65 years	637 (17.4)	7.1 (5.0, 9.7)	<0.001	14.6 (11.2, 17.8)	0.03	78.2 (72.4, 82.7)	0.005
BMI							
< 25 kg/m^2^	557 (15.2)	9.1 (6.2, 12.4)		16.7 (13.2, 20.3)		74.4 (67.3, 79.6)	
≥25 and < 30 kg/m^2^	1,236 (33.7)	8.4 (5.8, 11.5)		15.0 (11.6, 18.5)		76.3 (70.5, 81.3)	
≥30 and < 35 kg/m^2^	1,057 (28.9)	7.5 (5.5, 10.5)		13.3 (10.5, 16.5)		78.9 (73.3, 83.3)	
≥35 kg/m^2^	813 (22.2)	7.1 (5.2, 9.8)	<0.001	11.9 (9.1, 14.8)	<0.001	80.8 (75.9, 85.3)	<0.001
Study center							
Arizona	1,281 (35.0)	8.6 (6.1, 11.5)		13.3 (10.4, 16.4)		78.1 (72.6, 82.5)	
Oklahoma	1,141 (31.2)	6.6 (4.6, 9.3)		13.4 (10.2, 16.7)		79.6 (74.0, 84.3)	
North or South Dakota	1,241 (33.9)	8.4 (6.1, 11.7)	<0.001	15.3 (11.9, 19.2)	<0.001	75.9 (69.6, 81.1)	<0.001
Smoking status							
Never	1,177 (32.1)	7.4 (5.3, 10.1)		13.3 (10.1, 16.3)		79.0 (73.9, 83.5)	
Former	1,240 (33.9)	7.6 (5.4, 10.5)		13.6 (10.8, 17.1)		78.4 (72.8, 83.2)	
Current	1,246 (34.0)	9.0 (6.1, 12.2)	<0.001	15.2 (11.6, 19.0)	<0.001	75.8 (69.2, 81.3)	<0.001
Drinking status							
Never	575 (15.7)	7.1 (5.2, 9.8)		13.5 (10.6, 16.6)		79.2 (73.6, 83.3)	
Former	1,517 (41.4)	7.6 (5.4, 10.8)		13.9 (10.8, 17.3)		78.1 (72.4, 83.0)	
Current	1,571 (42.9)	8.4 (5.9, 11.7)	<0.001	14.2 (10.8, 18.0)	0.008	77.0 (70.8, 82.2)	<0.001
High school completion							
Yes	1,793 (47.5)	7.7 (5.5, 10.8)		13.9 (10.7, 17.3)		78.0 (72.3, 83.0)	
No	1,924 (52.5)	8.3 (5.7, 11.2)	0.002	13.9 (10.8, 17.7)	0.28	77.6 (71.7, 82.5)	0.03

*P values are from Kruskal-Wallis tests.

Arsenic % species must sum to 100% within each person.

In crude analyses, %iAs and %MMA were inversely related to body mass index (Spearman correlations -0.16, -0.32, respectively), percent body fat (-0.25, -0.35), and waist circumference (-0.15, -0.29), and positively related to fat free mass (0.15, 0.07) (Table 1). %DMA was positively related to body mass index (0.28), % body fat (0.35) and waist circumference (0.27), and inversely related to fat free mass (-0.12) (Table 1). All these Spearman correlations were significant at p < 0.0001. The correlations between fat free mass and %iAs, %MMA and %DMA were confounded by sex, and in analyses using sex-specific quartiles of fat free mass the crude associations were in the same direction as for the other measures of body composition (Table 3, Table 4).

**Table 3:** **Difference** (**95**% **CI**) **of mean % arsenic species in urine by body composition measure categories**

	**% iAs**		**% MMA**		**% DMA**	
	**Unadjusted**	**Adjusted**	**Unadjusted**	**Adjusted**	**Unadjusted**	**Adjusted**
Body mass index						
< 25 kg/m^2^	0.0 (referent)	0.0 (referent)	0.0 (referent)	0.0 (referent)	0.0 (referent)	0.0 (referent)
≥ 25, < 30 kg/m^2^	-0.8 (-1.3, -0.3)	-0.7 (-1.2, -0.3)	-1.5 (-2.0, -1.0)	-1.4 (-1.9, -1.0)	2.3 (1.5, 3.2)	2.2 (1.4, 3.0)
≥ 30, < 35 kg/m^2^	-1.8 (-2.3, -1.3)	-1.6 (-2.0, -1.1)	-3.1 (-3.6, -2.6)	-2.8 (-3.3, -2.3)	4.9 (4.0, 5.7)	4.3 (3.5, 5.1)
≥ 35	-2.3 (-2.8, -1.7)	-1.7 (-2.2, -1.2)	-4.8 (-5.4, -4.3)	-4.0 (-4.5, -3.5)	7.1 (6.2, 8.0)	5.7 (4.8, 6.5)
P for trend	< 0.001	<0.001	<0.001	<0.001	<0.001	<0.001
% Body fat						
Quartile 1	0.0 (referent)	0.0 (referent)	0.0 (referent)	0.0 (referent)	0.0 (referent)	0.0 (referent)
Quartile 2	-0.6 (-1.1, -0.2)	-0.6 (-1.0, -0.2)	-1.2 (-1.7, -0.8)	-1.1 (-1.6, -0.7)	1.9 (1.1, 2.7)	1.7 (1.0, 2.4)
Quartile 3	-0.9 (-1.4, -0.5)	-0.8 (-1.2, -0.4)	-1.9 (-2.4, -1.4)	-1. 7 (-2.1, -1.2)	2.8 (2.1, 3.6)	2.5 (1.8, 3.2)
Quartile 4	-1.2 (-1.6, -0.7)	-1.2 (-1.6, -0.7)	-3.0 (-3.4, -2.5)	-2.6 (-3.1, -2.2)	4.1 (3.4, 4.9)	3.8 (3.1, 4.5)
P for trend	<0.001	<0.001	<0.001	<0.001	<0.001	<0.001
Fat free mass						
Quartile 1	0.0 (referent)	0.0 (referent)	0.0 (referent)	0.0 (referent)	0.0 (referent)	0.0 (referent)
Quartile 2	-0.8 (-1.2, -0.3)	-0.8 (-1.2, -0.4)	-1.2 (-1.7, -0.7)	-1.2 (-1.7, -0.8)	2.0 (1.2, 2.7)	2.0 (1.3, 2.7)
Quartile 3	-0.6 (-1.1, -0.2)	-0.7 (-1.1, -0.2)	-1.5 (-2.0, -1.1)	-1.6 (-2.0, -1.1)	2.2 (1.4, 2.9)	2.2 (1.5, 2.9)
Quartile 4	-1.5 (-1.9, -1.0)	-1.4 (-1.9, -1.0)	-2.9 (-3.4, -2.5)	-2.8 (-3.3, -2.3)	4.4 (3.6, 5.2)	4.2 (3.5, 5.0)
P for trend	<0.001	<0.001	<0.001	<0.001	<0.001	<0.001
Waist circumference						
Quartile 1	0.0 (referent)	0.0 (referent)	0.0 (referent)	0.0 (referent)	0.0 (referent)	0.0 (referent)
Quartile 2	-0.8 (-1.2, -0.3)	-0.8 (-1.2, -0.4)	-1.2 (-1.7, -0.7)	-1.2 (-1.7, -0.8)	2.0 (1.2, 2.8)	2.0 (1.3, 2.7)
Quartile 3	-1.2 (-1.7, -0.8)	-1.2 (-1.6, -0.8)	-2.3 (-2.7, -1.8)	-2.2 (-2.6, -1.7)	3.5 (2.7, 4.2)	3.3 (2.6, 4.1)
Quartile 4	-1.6 (-2.0, -1.2)	-1.6 (-2.1, -1.2)	-3.4 (-3.9, -3.0)	-3.2 (-3.6, -2.7)	5.0 (4.3, 5.8)	4.8 (4.1, 5.5)
P for trend	<0.001	<0.001	<0.001	<0.001	<0.001	<0.001

Adjusted linear regression models control for age, sex, specific gravity-corrected arsenic, education, and drinking and smoking status.

Quartiles for waist circumference, % body fat and fat free mass use sex-specific cutpoints.

**Table 4:** **Difference** (**95**% **CI**) **of % arsenic species in urine by one unit increase in body composition measure categories in unadjusted models and in models adjusted for other measures of body composition**

	**% MMA by**				**% DMA by**			
	**BMI**	**% body fat**	**fat free mass**	**WC**	**BMI**	**% body fat**	**fat free mass**	**WC**
*Unadjusted models*								
Measure	-1.6 (-1.8, -1.4)	-1.0 (-1.1, -0.8)	-0.9 (-1.1, -0.8)	-1.1 (-1.3, -1.0)	2.4 (2.1, 2.6)	1.3 (1.1, 1.6)	1.3 (1.1, 1.6)	1.7 (1.4, 1.9)
*Models with multiple body composition measures*								
BMI, % body fat	-1.8 (-2.0, -1.5)	0.2 (0.0, 0.4)	-	-	2.7 (2.4, 3.1)	-0.4 (-0.8, -0.1)	-	-
BMI, fat free	-1.7 (-1.9, -1.5)	-	0.1 (-0.1, 0.3)	-	2.5 (2.1, 2.9)	-	-0.2 (-0.5, 0.2)	-
BMI, WC	-1.7 (-1.9, -1.4)	-	-	0.1 (-0.2, 0.3)	2.5 (2.1, 3.0)	-	-	-0.2 (-0.6, 0.2)
% Fat, fat free	-	-0.7 (-0.9, -0.6)	-0.7 (-0.8, -0.5)	-	-	1.0 (0.7, 1.2)	1.0 (0.7, 1.2)	-
% Fat, WC	-	-0.3 (-0.5, -0.1)	-	-0.9 (-1.1, -0.7)	-	0.4 (0.0, 0.7)	-	1.4 (1.1, 1.7)
BMI, % fat, fat free	-1.9 (-2.2, -1.6)	0.2 (0.0, 0.5)	0.1 (-0.1, 0.4)	-	3.0 (2.5, 3.5)	-0.5 (-0.9, -0.2)	-0.3 (-0.6, 0.0)	-
BMI, % fat, WC	-1.8 (-2.1, -1.5)	0.2 (0.0, 0.4)	-	0.0 (-0.3, 0.3)	2.8 (2.3, 3.2)	-0.4 (-0.8, -0.1)	-	0.0 (-0.4, 0.4)
% fat, fat free, WC	-	-0.4 (-0.6, -0.2)	-0.4 (-0.6, -0.2)	-0.6 (-0.9, -0.4)	-	0.5 (0.1, 0.8)	0.6 (0.2, 0.9)	1.0 (0.6, 1.4)
All	-1.9 (-2.2, -1.5)	0.3 (0.0, 0.5)	0.2 (-0.1, 0.4)	-0.1 (-0.3, 0.2)	3.0 (2.4, 3.5)	-0.6 (-1.0, -0.2)	-0.3 (-0.7, 0.0)	0.1 (-0.3, 0.6)

*BMI*: body mass index; *WC*: waist circumference.

Body composition measures were categorized as in Table 3 and entered into the model as ordinal.

Increasing body mass index categories remained associated with lower mean %iAs (-1.7, comparing BMI ≥ 35 to BMI < 25, with 95% CI: -2.2, -1.2) and %MMA (-4.0, 95% CI: -4.5, -3.5) and higher mean %DMA (+5.7, 95% CI: 4.8, 6.5) after adjustment for arsenic exposure and demographics (Table 3). Similar dose responses were observed for % body fat, fat free mass, and waist circumference when analyzed in separate models. In models adjusting for other measures of body composition at the same time, higher body mass index remained associated with lower %iAs and %MMA and higher %DMA. For waist circumference, the association with % arsenic species disappeared when adjusted for body mass index but remained when adjusting for % body fat and fat free mass. For % body fat and fat free mass, the associations disappeared when adjusting for body mass index or waist circumference, and were attenuated when adjusting for each other.

**Figure 1:**
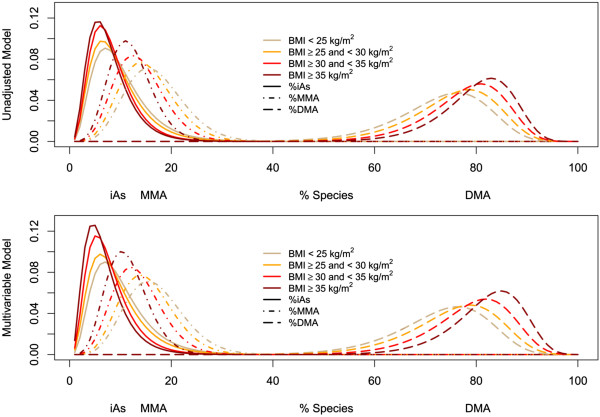
**Maximum likelihood estimates of generalized gamma models for % arsenic species by body composition measures.** The upper panel shows the crude associations of each % species with body mass index categories. The lower panel shows the residual association adjusting for categories of other body composition measures. Models allowed flexibility in location (β) and scale (σ). The % arsenic species are labeled at the marginal medians for each arsenic species.

**Figure 2:**
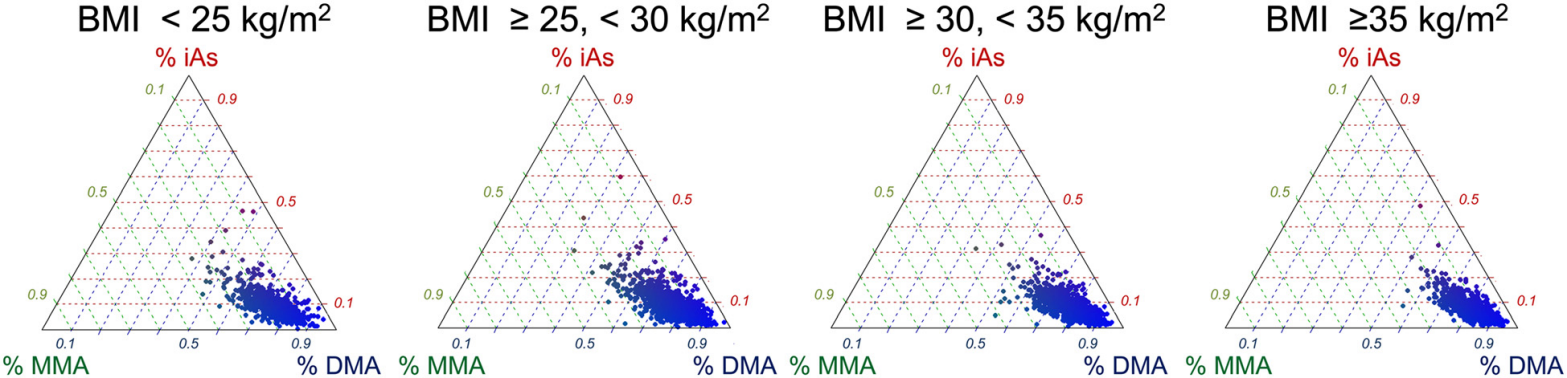
**Multivariate associations of body mass index and % arsenic species in urine using Dirichlet regression.** The % arsenic species (% iAs, % MMA and % DMA) lie on a simplex. As body mass increases, overall metabolism shifts to greater % DMA, and the metabolism profiles are less heterogeneous across individuals within the same body mass index stratum.

Our sensitivity analyses using generalized gamma regression, beta regression and Dirichlet regression were consistent with the findings obtained by linear regression (Figure 1, Additional file 3: Appendix 3). Both generalized gamma and beta regression models allowed us to evaluate differences in % arsenic species variability with increasing body mass index, showing that variability decreased with increasing body mass index categories (Figure 1). The shift of the population to more %DMA and less heterogeneity across individuals with increasing body mass index was also apparent from the multivariate Dirichlet analysis of all % arsenic species biomarkers (Figure 2). Results for each biomarker’s relationship with body mass index were similar in univariate beta regression and multivariate Dirichlet regression (Additional file 3: Appendix 3).

## Conclusion

Body mass index, % body fat, fat free mass and waist circumference were associated with lower %iAs, lower %MMA, and higher %DMA in urine of the Strong Heart Study participants. The associations remained similar after adjustment for age, sex, study center, education, drinking status, smoking status and urine arsenic levels. In analyses that adjusted for other measures of body composition, body mass index remained associated with % arsenic species while waist circumference remained associated when adjusted for % body fat or fat free mass but not when adjusted for body mass index. These findings indicate that body size is associated with % arsenic species, with higher body size being related to higher % DMA in urine. It is unclear, however, if the associations observed are due to adiposity, fat free mass, body size, or some other construct. Information bias from measurement error and correlation across body composition variables could explain the findings of models that adjust for multiple body composition variables. At first glance, the results of Table 4 might be interpreted as an indication that body size, as measured by body mass index, rather than adiposity or fat free mass, is what drives the association with arsenic metabolism. However, body mass index is likely the most precisely measured measure of body composition of those evaluated in this study, followed by waist circumference [53], which in turn is likely more precise than the two measures obtained from impedance, % fat mass and fat free mass [54]. In multivariable models, it is possible to transfer the association from a casual exposure to a better measured co-exposure that is not necessarily causally related to the outcome when there are positive correlation in exposures’ true values but negative correlations in measurement errors [55]. Thus, it is plausible that the consistently strong associations found with body mass index may be due to body mass index acting as a “sponge” for associations with other variables. To further disentangle the association of body composition measures with arsenic metabolism, it would be helpful to have multiple measures from the same visit of body mass index, % body fat, fat free mass and waist circumference to obtain estimates of the test-retest reliability of each measure in this population and more explicitly model the measurement error dependence.

Despite the challenges posed by measurement error for understanding the etiologic basis for the association, body mass index was cross-sectionally associated with the pattern of arsenic metabolites in urine. This association might be related to adiposity. Previous studies have shown that %DMA increases as women progress through pregnancy [56,57]. While the shifts are similar to those observed with increased body size in our study, it is unknown if those changes in arsenic metabolism during pregnancy are related to the concomitant gain in maternal body fat and body size [58,59]. Arsenic has been related to adiposity and to adipocyte differentiation in experimental models [60-63], although to our knowledge the roles of arsenic metabolism processes have not been evaluated. The association between body mass index and % arsenic metabolites may also be related to muscle mass. Creatinine, a break-down product of creatine phosphate in muscle is generally produced at a constant rate depending on muscle mass [64]. Creatinine may be a surrogate for several key mediators of the arsenic metabolism process as both arsenic metabolism [65] and creatine synthesis [66] use S-adenosyl methionine (SAM) as the methyl-donor. During methylation, SAM generates homocysteine which needs to be remethylated using folate and vitamin B12 [67] in order to be used again for methylation. Alternatively, homocysteine may form glutathione which is also important for arsenic species reduction and cellular efflux [67,68]. Experimental models are needed to evaluate if the connection between arsenic metabolism and measures of body composition are related to adiposity or to muscle mass.

We also found that those with higher body mass index had less variability in their distribution of %DMA than those with lower body mass index. To our knowledge this has never been explicitly discussed in previous studies. However, this same pattern is visible in published histograms of %DMA compared over the development of pregnancy [56,57]. It has been shown that as women progress through pregnancy, they have higher %DMA in urine and more peaked distributions of %DMA [56,57], much like the patterns seen across body size groups in our study. We suspect that the reduced variability with increasing adiposity or with the progression of pregnancy may reflect a constraint on maximum possible %DMA. It would be interesting to evaluate if the pattern observed during pregnancy is related to increased adiposity.

Our study has a number of strengths and limitations. Strengths include the large sample size, multiple measures of body size, and the laboratory techniques for arsenic speciation, characterized by low limits of detection. Similar to other studies on arsenic metabolism, we are limited by the absence of data on arsenic species intake and routes of exposure. However, it is likely that the primary arsenic exposure for our population is inorganic arsenic from drinking water and that MMA and DMA observed in urine are mainly from metabolized inorganic arsenic [49]. The lack of data on arsenic species concentrations within the body, for instance in blood or adipose tissue, also limits pharmacokinetic inferences. This is a cross-sectional study, and the direction of the association is uncertain. Study designs with longitudinal data might enable evaluating the direction of the association and the impact of changes in body mass index in arsenic metabolism. Genetic advances characterizing arsenic metabolism could potentially enable Mendelian randomization studies of arsenic metabolism and adiposity [69]. Unmeasured confounding is possible. However, since these are large-magnitude associations, the unmeasured variables would need to be highly aliased with body size and with arsenic metabolite proportions to explain the observed associations. Fat-soluble chemicals for instance are strongly related to adiposity, but their relationship to arsenic metabolism is unknown.

## Conclusions

Body mass index was associated with arsenic metabolite distributions in urine even after adjustment for other measures of body composition. These findings suggest that increased body size is related to higher % DMA in urine, although we could not reach firm conclusions whether the association with body mass index is related to adiposity or fat free body mass, due to the potential impact of measurement error in models with multiple measures of body composition. Potentially, the association with body mass index could also be related to body size *per se*, rather than only adiposity or fat free mass. Experimental research can potentially advance our understanding of the relationship between body composition and arsenic metabolism. Prospective epidemiologic studies are also needed to evaluate the direction of the relationship between arsenic metabolism and body mass index. Inter-individual differences in arsenic metabolism may be important for population-level variation in arsenic susceptibility. Given the association between body mass index and arsenic metabolism, epidemiologic studies of arsenic health effects should examine whether body mass index may be a modifier of arsenic disease risks.

## Abbreviations

BMI: Body mass index; iAs: Inorganic arsenic; MMA: Methylarsonate (a methylated arsenic species); DMA: Dimethylarsinate (a methylated arsenic species); %iAs: Proportion of inorganic arsenic contributing to sum iAs + MMA + DMA in urine; %MMA: Proportion of methylarsonate contributing to sum iAs + MMA + DMA in urine; %DMA: Proportion of dimethylarsinate contributing to sum iAs + MMA + DMA in urine; SAM: S-adenosyl methionine.

## Competing interests

The authors declare that they have no competing interests.

## Author’s contributions

MOG drafted the manuscript, conducted statistical analyses, and contributed to the discussion. BVH, JGU, and YZ provided data, edited the manuscript and contributed to the discussion. KAF and WG conducted laboratory analyses, edited the manuscript and contributed to the discussion. CMC and YZ provided statistical support and contributed to the discussion. EKS and EG edited the manuscript and contributed to the discussion. ANA drafted the manuscript, supervised statistical analyses, and contributed to the discussion. All authors read and approved the final manuscript.

## Supplementary Material

Additional file 1: Appendix 1 Strong Heart Study population and analysis sample. Click here for file

Additional file 2: Appendix 2 Histograms of % arsenic species. The solid line over each histogram represents the maximum likelihood estimate of the corresponding generalized gamma model. Click here for file

Additional file 3: Appendix 3 Difference (95% CI) of mean % arsenic species in urine by body mass index categories using beta and Dirichlet regression models. Models for each separate % arsenic species biomarker allowed for flexibility in mean (μ) and dispersion (φ) parameters according to categories of body mass index. Click here for file
