# Supplementary material for: Body composition and arsenic metabolism: a cross-sectional analysis in the Strong Heart Study
Source: Environ Health. 2013 Dec 9;12:107. doi: 10.1186/1476-069X-12-107 (PMC3883520; doi:10.1186/1476-069X-12-107)
Supplement: Additional file 1: Appendix 1 — Strong Heart Study population and analysis sample. [file 1476-069X-12-107-S1.docx]

**Appendix 1:** Strong Heart Study population and analysis sample.

|  | Strong Heart Study (N= 4,549) | Study Sample (N=3,663) |
| --- | --- | --- |
| Age (years) | 56.3 (8.1) | 56.1 (8.0) |
| % Male | 40.6 | 41 |
| % Completed High School | 52.2 | 52.5 |
| % Ever Smokers | 67.7 | 67.9 |
| * Pack-Years Smoked | 16.3 (20.4) | 16.2 (20.4) |
| % Never Drinkers | 16.3 | 15.7 |
| % Former Drinkers | 42.3 | 41.4 |

Data are mean (SD) or %

*Pack-years smoked was measured among ever smokers, observed N=2,897 in the overall SHS database and N=2,356 in the study sample.
