# Supplementary material for: Body composition and arsenic metabolism: a cross-sectional analysis in the Strong Heart Study
Source: Environ Health. 2013 Dec 9;12:107. doi: 10.1186/1476-069X-12-107 (PMC3883520; doi:10.1186/1476-069X-12-107)
Supplement: Additional file 2: Appendix 2 — Histograms of % arsenic species. The solid line over each histogram represents the maximum likelihood estimate of the corresponding generalized gamma model. [file 1476-069X-12-107-S2.docx]

**Appendix 2**. **Histograms of % arsenic species.** The solid line over each histogram represents the maximum likelihood estimate of the corresponding generalized gamma model.

**
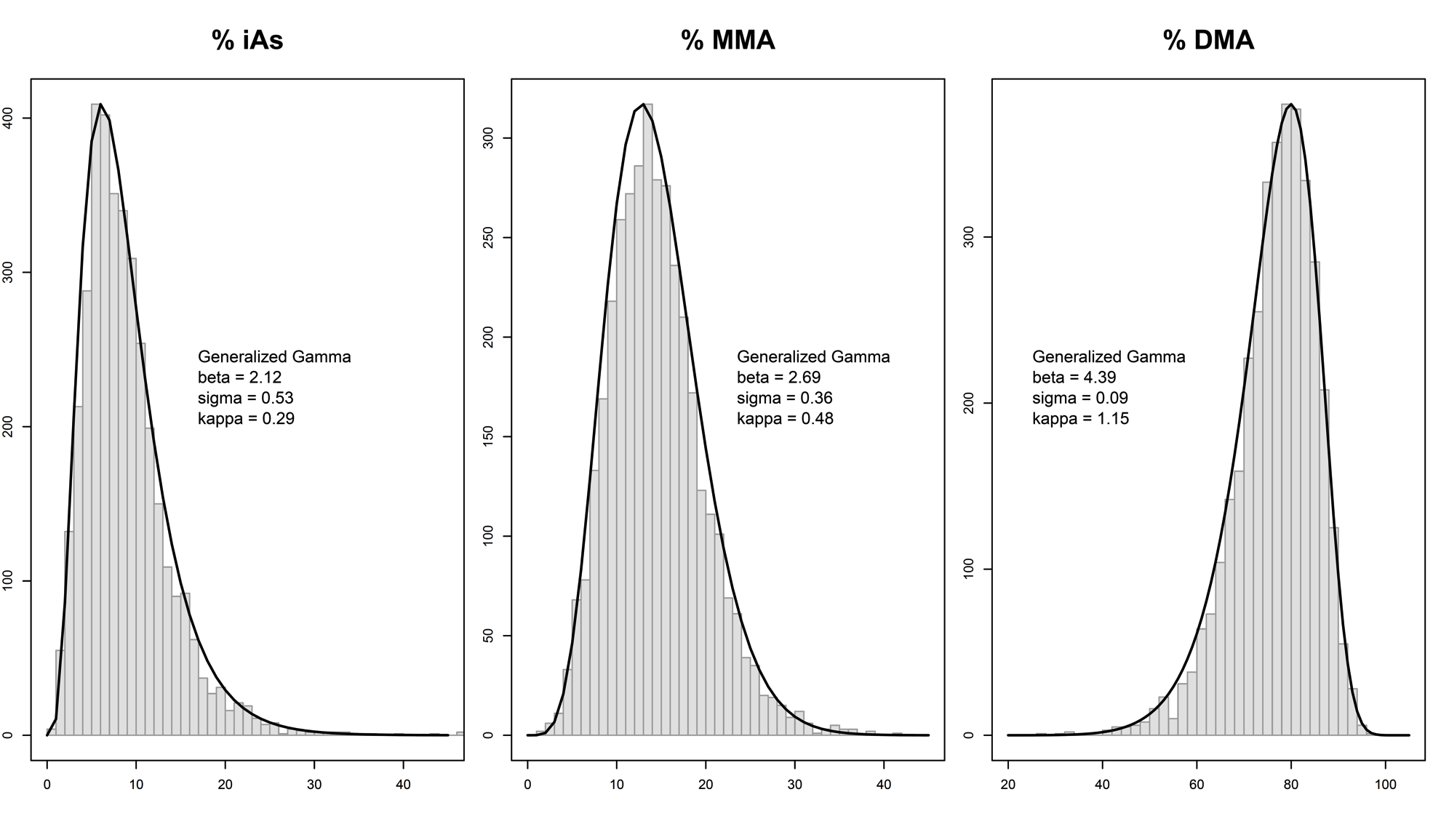
**
