# Supplementary material for: Body composition and arsenic metabolism: a cross-sectional analysis in the Strong Heart Study
Source: Environ Health. 2013 Dec 9;12:107. doi: 10.1186/1476-069X-12-107 (PMC3883520; doi:10.1186/1476-069X-12-107)
Supplement: Additional file 3: Appendix 3 — Difference (95% CI) of mean % arsenic species in urine by body mass index categories using beta and Dirichlet regression models. Models for each separate % arsenic species biomarker allowed for flexibility in mean (μ) and dispersion (φ) parameters according to categories of body mass index. [file 1476-069X-12-107-S3.docx]

**Appendix 3. Difference (95% CI) of mean % arsenic species in urine by body mass index categories using beta and Dirichlet regression models**. Models for each separate % arsenic species biomarker allowed for flexibility in mean (μ) and dispersion (φ) parameters according to categories of body mass index.

|  | %iAs | | %MMA | %DMA |  |
| --- | --- | --- | --- | --- | --- |
| *Beta Regression* |  | |  |  |  |
| Body Mass Index |  | |  |  |  |
| < 25 kg/m^2^ | 0.0 (referent) | | 0.0 (referent) | 0.0 (referent) |  |
| ≥ 25, < 30 kg/m^2^ | -0.8 (-1.2, -0.3) | | -1.4 (-1.8, -0.9) | 2.1 (1.4, 2.9) |  |
| ≥ 30, < 35 kg/m^2^ | -1.7 (-2.1, -1.2) | | -2.8 (-3.3, -2.4) | 4.6 (3.8, 5.3) |  |
| ≥ 35 kg/m^2^ | -2.1 (-2.5, -1.7) | | -4.5 (-4.9, -4.0) | 6.7 (5.9, 7.4) |  |
|  |  | |  |  |  |
| *Dirichlet Regression* | |  |  |  | |
| Body Mass Index |  | |  |  | |
| < 25 kg/m^2^ | 0.0 (referent) | | 0.0 (referent) | 0.0 (referent) | |
| ≥ 25, < 30 kg/m^2^ | -0.8 (-1.2, -0.3) | | -1.4 (-1.9, -0.8) | 2.1 (1.5, 2.8) | |
| ≥ 30, < 35 kg/m^2^ | -1.6 (-2.1, -1.2) | | -3.0 (-3.5, -0.02) | 4.6 (3.9, 5.2) | |
| ≥ 35 kg/m^2^ | -2.2 (-2.6, -1.8) | | -4.5 (-5.1, -4.0) | 6.7 (6.1, 7.4) | |
